# Transcriptome Analysis of Male and Female Mature Gonads of Silver Sillago (*Sillago sihama*)

**DOI:** 10.3390/genes10020129

**Published:** 2019-02-11

**Authors:** Changxu Tian, Zhiyuan Li, Zhongdian Dong, Yang Huang, Tao Du, Huapu Chen, Dongneng Jiang, Siping Deng, Yulei Zhang, Saetan Wanida, Hongjuan Shi, Tianli Wu, Chunhua Zhu, Guangli Li

**Affiliations:** Guangdong Research Center on Reproductive Control and Breeding Technology of Indigenous Valuable Fish Species, Key Laboratory of Marine Ecology and Aquaculture Environment of Zhanjiang, Key Laboratory of Aquaculture in South China Sea for Aquatic Economic Animal of Guangdong Higher Education Institutes, Fisheries College, Guangdong Ocean University, Zhanjiang 524088, China; tiancxgdou@163.com (C.T.); G_YL903@163.com (Z.L.); dzhd888@163.com (Z.D.); zjouhy@126.com (Y.H.); zjhddutao@163.com (T.D.); chpjwx@163.com (H.C.); jdn1987@163.com (D.J.); sipingdeng@126.com (S.D.); yuleizhang88@163.com (Y.Z.); wanidamnk62@gmail.com (S.W.); shihongjuan1990@163.com (H.S.); wtianli@163.com (T.W.); zhu860025@163.com (C.Z.)

**Keywords:** *Sillago sihama*, transcriptome, gonadal development and gametogenesis, differentially expressed genes

## Abstract

Silver sillago (*Sillago sihama*) is an emerging commercial marine aquaculture species in China. To date, fundamental information on *S. sihama*, such as genomic information, is lacking, and no data are available on the gonad transcriptome of *S. sihama*. Here, the first gonadal transcriptomes of *S. sihama* have been constructed and genes potentially involved in gonadal development and reproduction identified. Illumina sequencing generated 60.18 million clean reads for the testis and 59.10 million for the ovary. All reads were assembled into 74,038 unigenes with a mean length of 1,004 bp and N50 value of 2,190 bp. Among all the predictable unigenes, a total of 34,104 unigenes (46%) were searched against multiple databases, including 33,244 unigenes annotated in the RefSeq Non- Redundant database at NCBI, and 28,924 in Swiss-Prot. By comparing the ovary and testis, 35,367 unigenes were identified as being differentially expressed between males and females, of which 29,127 were upregulated in the testis and 6,240 were upregulated in the ovary. Numerous differentially expressed genes (DEGs) known to be involved in gonadal development and gametogenesis were identified, including *amh*, *dmrt1*, *gsdf*, *cyp19a1a*, *gnrhr*, and *zps*. Using gene ontology (GO) and Kyoto Encyclopedia of Genes and Genomes (KEGG) enrichment analyses, the top 20 KEGG pathways with highest number of DEGs were found to be involved in regulating gonadal development and gametogenesis in *S. sihama*. Moreover, 22,666 simple sequence repeats (SSRs) were identified in 14,577 SSR-containing sequences. The findings provide a valuable dataset for future functional analyses of sex-associated genes and molecular marker assisted selection in *S. sihama.*

## 1. Introduction

Fishes are one of the most abundant vertebrates on Earth, including approximately 30,000 species [1]. Sex determination and evolution in fish is more complex than in other species and can be influenced by genetic and environmental factors [2,3]. To date, several master sex-determining genes have been reported in multiple fish species [4], including *dmy* (Y-specific DM-domain) in medaka, *sdY* (sexually dimorphic on the Y chromosome) in rainbow trout, *amhr2* (anti-Müllerian hormone receptor type II) in fugu, *amhy* (anti-Müllerian hormone) in Patagonian pejerrey, *gsdf* (gonadal soma-derived growth factor on the Y chromosome) in rainbow trout and *dmrt1* (doublesex and mab-3 related transcription factor 1) in the half-smooth tongue sole. Besides these master sex determining genes, some conserved genes (such as *sox9*, *fox2*, *fox3*, *sf1*, and *cyp19*) control sexual development and differentiation, mainly via the action of steroid hormones [4,5]. In addition, many biological pathways such as the Wnt signaling pathway, estrogen signaling pathway and transforming growth factor β (TGF-β) signaling pathway, also play essential roles in sex determination and reproduction [6]. To better understand sexual regulatory mechanisms in fish, it is necessary to explore sex-related genes in more detail.

Transcriptome sequencing provides a general representation of the genes that are expressed in specific tissues or organs [7] and has been widely applied to identifying sex-related genes and associated regulatory mechanisms, due to its advantages of high throughput and low cost. A set of sex-related genes or pathways has been identified by gonad transcriptomes in numerous aquaculture species, including the spotted knifejaw [6], Japanese scallop [8], olive flounder [9] and channel catfish [10].

As the most widespread species of Sillaginidae, the silver whiting *Sillago sihama* is naturally distributed in the Indo-Pacific region [11]. Due to its high meat quality, taste and year-round breeding pattern, it is emerging as a commercial marine aquaculture species in China. However, despite its economic importance, studies on this species have been limited to a description of its reproductive biology, artificial breeding, feeding habits and population genetics [12,13,14,15]. To date, fundamental details, such as genomic information, on *S. sihama* are lacking and no data are available on the gonad transcriptome of *S. sihama*. In this study, we performed the first gonadal transcriptome of *S. sihama* and annotated genes potentially involved in gonadal development and reproduction. This transcriptome dataset will provide a foundational understanding for further research on the regulatory mechanisms of sex determination and development in *S. sihama*.

## 2. Materials and Methods

### 2.1. RNA Extraction, Complementary DNA Library Construction and Illumina Sequencing

*Sillago sihama* reaches sexual maturity at one year [15]. Two two-year-old adult *S. sihama* individuals (male: body length 22 cm, body weight 87 g; female: body length 27 cm, body weight 108 g) were obtained from the East Island in Zhanjiang, China (21°00′33.00″N, 110°23′5.99″E). Gender was identified by morphological observation of the gonads. Samples of the testis and ovary were rapidly sampled in RNAlater stabilization reagent and stored at -80 °C. Then, total RNA of the mature gonads (male, named Ss_M; female, named Ss_F) was extracted using Trizol reagent (Invitrogen, Carlsbad, CA, USA) according to the manufacturer’s instructions. All experimental protocols were approved by the Animal Research and Ethics Committee of Guangdong Ocean University (NIH Pub. No. 85–23, revised 1996).

Complementary DNA (cDNA) libraries from one ovary and one testis were constructed separately with TrueSeq RNA Sample Prep Kit (Illumina, San Diego, CA, USA) according to the manufacturer’s protocol. Messenger RNA (mRNA) was enriched from total RNA by Oligo-(dT) beads (Illumina), and fragments were subjected to end repair, adapter ligation and size selection by agarose gel electrophoresis, with suitable fragments selected as templates for PCR amplification. A 2100 Bioanaylzer (Agilent, Santa Clara, CA, USA) and StepOnePlus Real-Time PCR System (Thermo-Fisher Scientific, Santa Clara, CA, USA) were used for checking the quantity and quality of the sample library. RNA samples (displaying an RNA Integrity Number higher than 8) were selected for the preparation of sequencing libraries. The libraries were sequenced using an Illumina HiSeq^TM^ 2500 platform (v4 reagents) with a 150-bp paired-end strategy. All raw data have been submitted to the NCBI sequence read archive (SRA) under accession number PRJNA503317 (Male: SRR8143991; Female: SRR8143992).

### 2.2. De Novo Assembly and Functional Annotation

After removing adaptors, leading and trailing low quality or N bases (below quality three), scanning the read with a 4-base wide sliding window, cutting when the average quality per base dropped below 15, and dropping reads below 36 bases long, clean data was then de novo assembled using Trinity software (version 2.0.6) [16] with min_kemer_cov set to 3 and all other parameters set as default (scripts can be found at Data Supplementary Data S1). Contigs longer than 200 bp were filtered out for the following analysis after assembly. Unigenes were formed when contigs could not be further stretched on either end. These unigenes were further spliced by a *k*-mer cut-off value of 25 and then assembled to acquire maximum length nonredundant unigenes using TGICL clustering software (J. Craig Venter Institute, Rockville, MD, USA). To annotate all unigenes, the BLASTx program (version 2.2.23) (https://blast.ncbi.nlm.nih.gov/) with an *E*-value threshold of 1 × 10^−5^ was used to query against different databases: NCBI non-redundant protein (NR) database (https://www.ncbi.nlm.nih.gov/refseq/), the Swiss-Prot protein database (https://www.uniprot.org/), the Kyoto Encyclopedia of Genes and Genomes (KEGG) database (https://www.genome.jp/kegg/), and the eukaryotic orthologous group (KOG) database [17]. Gene ontology (GO) annotation and functional classification of unigenes were performed using Blast2GO software (https://www.blast2go.com/; version 4.4) and WEGO software (http://wego.genomics.org.cn/; version 1.0), respectively.

### 2.3. Single Sequence Repeat Locus Identification

MIcroSAtellite (MISA) software (http://pgrc.ipk-gatersleben.de/misa/misa.html; version 1.0) was applied for single sequence repeat (SSR) mining of all unigenes. The default parameters of mining were as follows: the minimum number of repeat units for di-, tri-, tetra-, penta- and hexa-nucleotide motifs were set as 6, 5, 4, 4 and 4, respectively. Primer 1.1.4 was then used to design primer pairs in the flanking regions of SSRs for subsequent validation.

### 2.4. Differential Gene Expression Analysis

High quality clean reads were mapped to the reference transcriptome using the short reads alignment tool Bowtie2 with default parameters. The reads per kilobase per million reads (RPKM) method [18] was used to measure gene expression levels of *S. sihama*. The edgeR package (version 3.4.3) [19] was used to identify differentially expressed genes (DEGs) between ovary and testis (scripts can be found at Supplementary Data S1). Unigenes with a fold change ≥ 4 and a false discovery rate (FDR) < 0.01 were considered as DEGs. The DEGs were then subjected to enrichment analysis of GO functions and KEGG pathways, and those with *p* ≤ 0.05 were considered significantly enriched.

### 2.5. Experimental Validation by Quantitative real-time Polymerase Chain Reaction

A total of 15 genes were selected for quantitative real-time polymerase chain reaction (qRT-PCR) validation and primers were designed by an online software tool Primer-BLAST [20]. qRT-PCR was performed on an ABI 7500 qPCR system (Life Technologies Inc., Carlsbad, CA, USA) using SYBR Green Real time PCR Master Mix (TaKaRa Biotechnology, Dalian, China) as per the following process: denaturation at 95 °C for 1 min, followed by 40 cycles of 95 °C (15 s), 60 °C (30 s), and 72 °C (45 s). The ribosomal protein L7 (*rpl7*) gene was used to normalize the expression values [21]. The 2^−ΔΔCt^ method was used to calculate relative expression levels of the target gene. All primers used in real-time PCR are shown in Table S1.

## 3. Results and Discussion

### 3.1. Illumina Sequencing and De Novo Assembly

The first gonad transcriptome in *S.sihama* will provide a valuable genomic resource for future studies on gonadal development and gametogenesis, and enrich candidate genes involved in these processes in marine fish. In total, 17.89 Gb bases of data were generated (63.69 million raw reads for the testis and 62.05 million raw reads for the ovary) (Table 1). After performing quality control, 60.18 million clean reads were retained for the testis and 59.10 million reads were retained for the ovary. A total of 74,038 unigenes were assembled by Trinity [16]. By categorizing both clusters and singletons as unigenes, a total of 74,038 unigenes were assembled by Trinity. The total number of unigenes looks much higher than commonly imagined, which might be due to the high heterozygosity and high repetition rate of the genome of Sillaginidae fishes [22]. Using the same assembly method, large numbers of unigenes were also found in crab [7] and shrimp [23], species whose genomes also have high heterozygosity and high repetition rates. The sequence length distribution of the unigenes ranged from 201 bp to 20,589 bp, with a mean length of 1,004 bp (Table 2). The number of unigenes that exceeded 1,000 bp was 19,591 (26.5%).

### 3.2. Sequence Annotation

**Figure 1:**
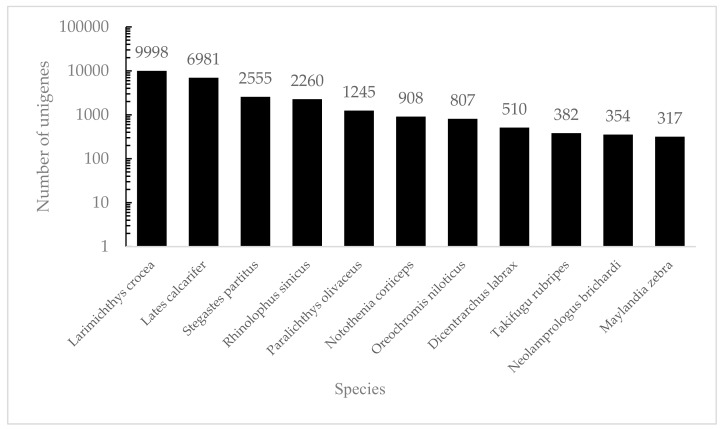
The species distribution of the results of NR annotation. Vertical axes: the number of annotated sequences matching each species. Horizontal axes: the distribution of top species that match the annotated sequences.

Among all the predictable unigenes, a total of 34,104 unigenes (46.07%) were searched against multiple databases, while 39,931 (53.93%) unigenes had no BLASTx hits. Among all the annotated unigenes, 33,244 unigenes matched against the NR database (97.48%) and 28,924 against Swiss-Prot (84.81%) (Table 2). These annotated unigenes provided the basis for further study on specific molecular processes in *S. sihama*. This may be related to the limited genomic information available for members of the Sillaginidae, or because these unannotated unigenes were specific sequences containing unknown protein domains, genes’ untranslated regions, or non-coding RNAs [23]. Based on the NR database, a total of 33,244 unigenes matched known sequences from 688 different species. Most unigene matches were to *Larimichthys crocea* (30.07%), *Lates calcarifer* (21.00%), *Stegastes partitus* (7.69%), *Rhinolophus sinicus* (6.80%) and *Paralichthys olivaceus* (3.75%) (Figure 1), for which genomic information is available.

**Figure 2:**
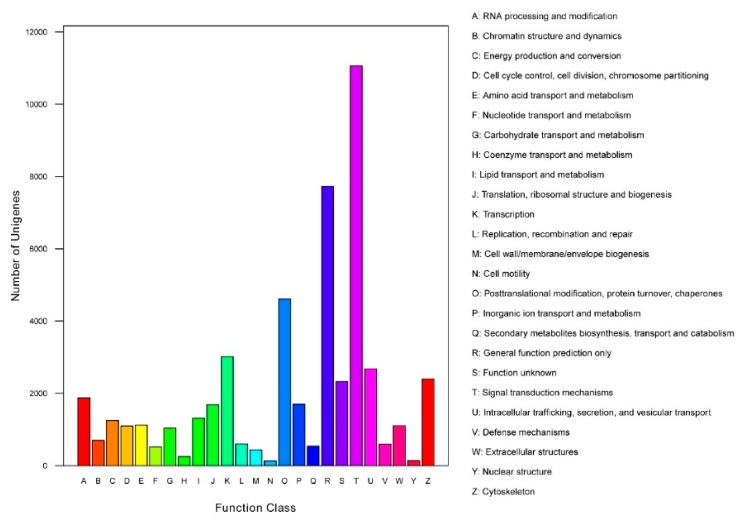
Clusters of KOG function classifications of all annotated unigenes in *S. sihama* gonadal transcriptome. Each function class is represented by a capital letter under the horizontal axis. The vertical axis denotes the number of unigenes in the corresponding function class.

**Figure 3:**
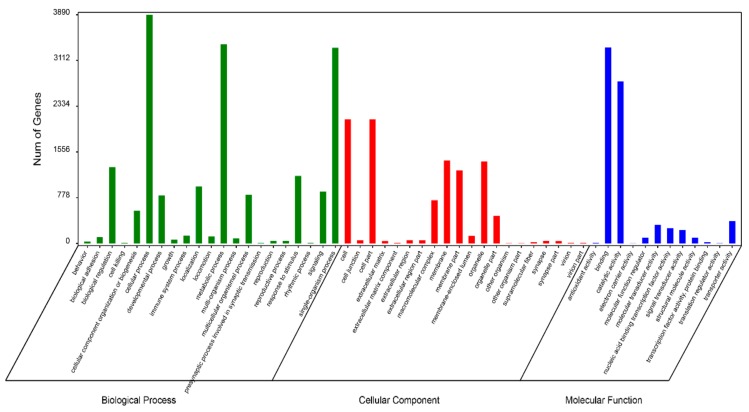
Functional annotation of all unigenes based on GO categorization. The X-axis and Y-axis indicate the GO functions and the number of unigenes with GO function, respectively.

Functional prediction and classification of the unigenes was conducted by searching the KOG and GO databases. In the KOG database, 23,064 unigenes were annotated and classified into 25 functional categories (Figure 2), the top three of which were: signal transduction mechanisms (11,063, 48.0%); general function prediction only (7733, 33.5%); and posttranslational modification, protein turnover, chaperones (4613, 20.0%). 6,938 unigenes were classified into three major GO categories (Figure 3). Among these functional groups, the terms “cellular process” (56.04%), “binding” (48.00%), and “cell” (30.38%) were dominant in the biological process, molecular function ontologies, and cellular component categories, respectively. Similar proportions were observed in previous transcriptome analyses of other fish [24,25,26], suggesting that the genes encoding these functions are highly conserved across species and are more easily annotated [23]. In this study, 9,806 unigenes were mapped to 237 KEGG pathways, and the number of unigenes in different pathways ranged from 1 to 2,377 (Table S2). A total of 24.24% of unigenes were mapped to ‘metabolic pathway’, indicating this pathway plays an important role in the development and function of silver sillago gonads. Additionally, we identified several KEGG pathways related to reproduction and development, such as the MAPK signaling pathway (508 unigenes), Wnt signaling pathway (316 unigenes), GnRH signaling pathway (229 unigenes) and oocyte meiosis (209 unigenes).

### 3.3. Differential Gene Expression Identification and Enrichment Analysis

**Figure 4:**
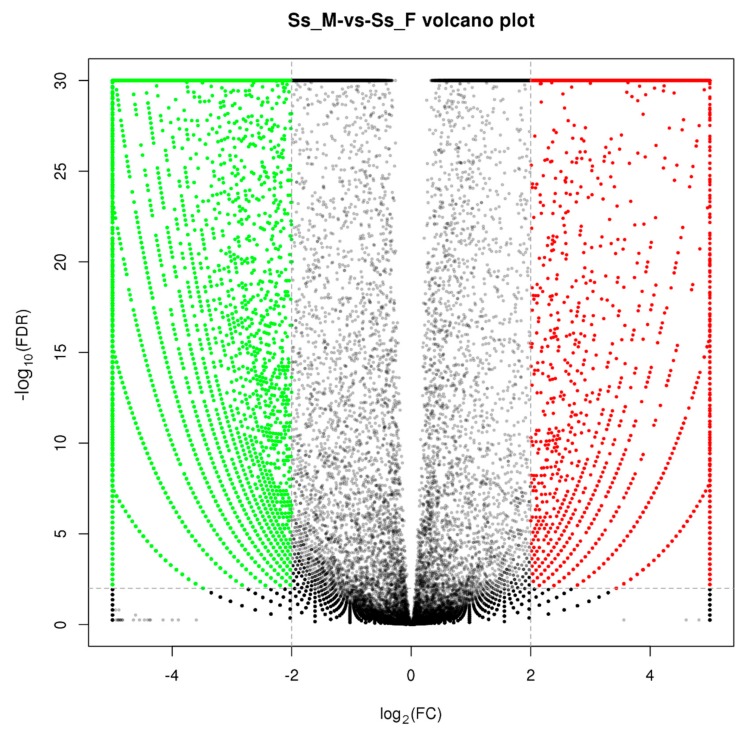
Volcano plot of differences in gene expression between testes and ovaries. Red: upregulated, represents ovary-biased genes; Green: downregulated, represents testis-biased genes.

In this study, 35,367 unigenes were found to be DEGs between the male and female (FDR ≤ 0.01 and log_2_FC ≥ 2) (Figure 4). A total of 13,324 (37.67%) DEGs were annotated, with 9,815 up- and 3,509 down-regulated genes in the male. Many male-biased unigenes and female-biased unigenes were identified in *S. sihama*, as in gonadal transcriptome analyses of other species [23,27], indicating that reciprocal antagonism between male-promoting and female-promoting pathways is a central feature underlying sexual development and maintenance in vertebrates.

**Figure 5:**
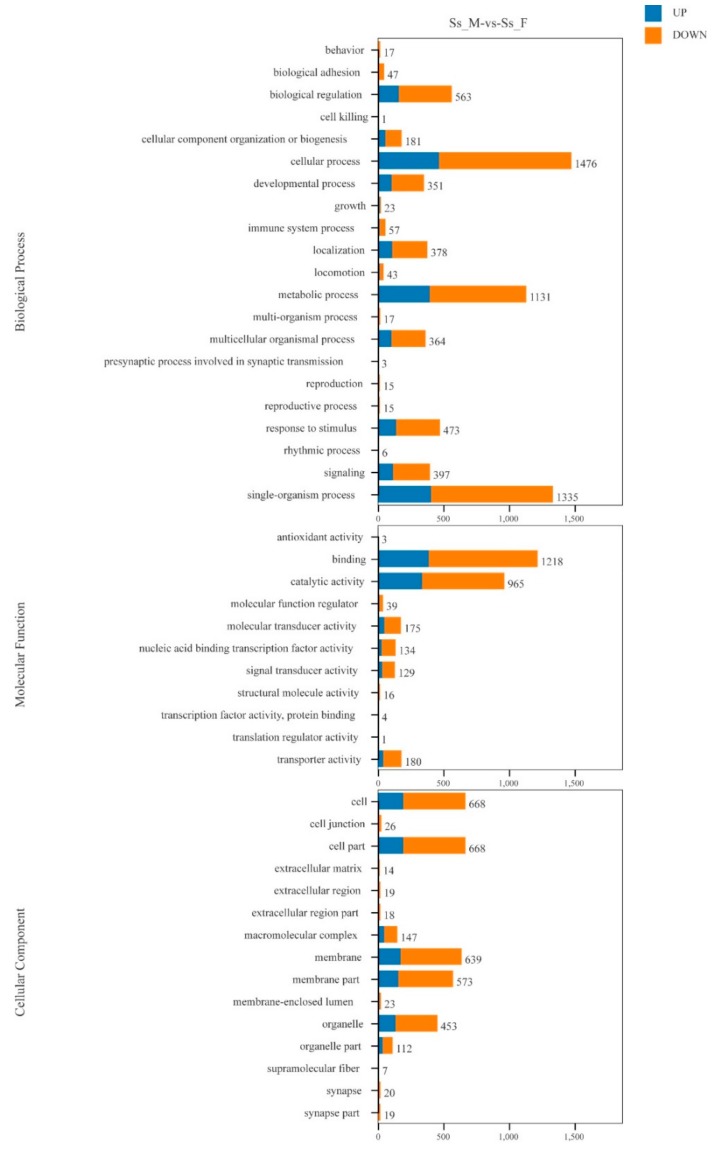
Distribution of differentially expressed genes (DEGs) among GO terms in biological process, cellular component, and molecular function.

GO annotation was performed to classify the 13,324 DEGs (Figure 5). At the biological process level, the GO terms “cellular process” (1476 unigenes), “single-organism process” (1335 unigenes) and “metabolic process” (1131 unigenes) included more DEGs. There are also more DEGs in the GO terms “binding” (1218 unigenes) and “catalytic activity” (965 unigenes) at the molecular functional level. At the cellular component level, more DEGs were seen in the GO terms “cell” (668 unigenes), “cell part” (668 unigenes), and “membrance” (639 unigenes). GO enrichment analysis showed that 106, 10, and 60 GO terms were significantly enriched in the categories “biological process”, “cellular component”, and “molecular function”, respectively (Table S3) (*p* < 0.05). Meanwhile, 3,323 DEGs were mapped to 217 KEGG pathways, and 27 significant KEGG pathways were obtained (with a threshold of *Q* value < 0.05, Table S4).

#### 3.3.1. Genes Related to Gonadal Development and Gametogenesis

Numerous differentially expressed genes known to be involved in sex control and gonadal development were identified in the *S. sihama* gonad transcriptome, including doublesex- and mab-3-related transcription factor 1 (*dmrt1*), anti-Müllerian hormone (*amh*), gonadal soma derived factor 1 (*gsdf*), p450 aromatase (*cyp19a1a*), gonadotrophin releasing hormone receptor (*gnrhr*), zona pellucida sperm-binding proteins (*zps*) and other potential candidate genes (Table 3). Among these candidate genes, several documented and important genes are described in further detail.

As a sex determining gene in fish, the *dmrt1* gene encodes a transcription factor that is essential for the maintenance of male-specified germ cells and testis differentiation [28]. Anti-Müllerian hormone (*amh*) has been identified in several fish species. As a member of the TGF-β family, *amh* is a central player in gonad development and a target of gonadotropic follicle-stimulating hormone (*fsh*) in several fish species [29,30]. In rainbow trout, *gsdf*, a new TGF-β superfamily member, was identified and found to regulate the proliferation of primordial germ cells (PGCs) and spermatogonia [31]. A previous study has shown that overexpression of *gsdf* induced testis differentiation in Nile tilapia females [32]. In this study, these sex-determining genes were identified and showed a male-biased pattern of expression in *S. sihama*.

Steroid hormones play vital roles in germ cells, regulating sexual differentiation and sexual behaviour patterns [33]. *Cyp11b* and *cyp21* play a key role in catalyzing the production of cortisol and are involved in sex steroid production [34]. Previous studies have shown that *cyp11b* mRNA was expressed at higher level in males than in females in several fish species, indicating *cyp11b* was involved in the regulation of testicular development [35,36]. The aromatase cytochrome P450 family 19 subfamily A member 1 (*cyp19a1a*) gene catalyzes a key step in the conversion of testosterone to estrogen [37]. Here, the high expression of steroid-metabolizing enzymes (*cyp11b*, *cyp17a1*, and *cyp27a1*) found in the testis, and *cyp19a1a* in the ovary (Table 3), indicated that steroid-metabolizing enzymes may also participate in the development of gonads and regulate the synthesis of steroid hormones in *S. sihama*.

Sox (Sry-type HMG box) proteins are involved in the regulation of fish sex determination and differentiation. *Sox6*, a SOX D transcription factor, was exclusively expressed in the testis and is involved in the later stages of spermatogenesis [38]. In mammals, SRY-related transcription factors from the group SOX E subfamily (*Sox8*, *Sox9* and *Sox10*) play important roles in the regulation of testis organogenesis and spermatogenesis [39]. In this study, *Sox6* and two SOX E genes (*Sox8* and *Sox10*) were expressed at higher levels in the male gonads than in the female gonads, suggesting that the SOX genes play key roles in testis development in *S. sihama*.

Perm acrosome membrane-associated protein 6 (*spaca6*), sperm-associated antigen 17 (*spag17*), spermatogenesis-associated protein 17 (*spata17*), spermatogenesis-associated protein 4 (*spata4*) and sperm flagellar protein 2 (*spef2*) were significantly more highly expressed in male compared to female gonads, and the expression difference might be associated with the spermatogenesis in silver sillago. *Spaca6* has been shown to be a sperm membrane component that mediates fusion between the ovum and the spermatozoa [40]. *Spag17*, *Spef2*, and *Sp17* have been shown to have essential functions in spermatogenesis [8].

The zona pellucida (ZP) is an extracellular glycoproteinaceous matrix shell that is involved in oocyte and gamete development. In fish, the expression level of *zp* genes’ mRNA is significantly increased during oogenesis, especially at the previtellogenic stage, which shows a higher expression level than at undeveloped stages [41]. Previous studies showed that the *zp2* gene played a key role in the early formation of the oocyte envelope and *zp3* acted as a major class of female-specific molecules for reproduction [42]. In this study, three ZP genes (*zp2*, *zp3*, and *zp4*) showed higher expression levels in the *S. sihama* ovary compared to the testis, suggesting that these oocyte-specific genes may also play important roles in folliculogenesis and reproduction.

A set of 15 selected genes was validated by qRT-PCR, including eight upregulated genes in the male (*amh*, *cyp11b*, *cyp27a1*, *dmrt1*, *dmrtb1*, *foxl1*, *gsdf*, *izumo1*), and seven upregulated genes in the female (*cyp19a1a*, *dmrt3*, *gnrhr2*, *igfbp1*, *spaca4*, *zp2*, *zp4*). The expression patterns of all genes indicated by qPCR analysis were similar to those indicated by RNA-seq (Table S5), indicating the reliability and accuracy of the transcriptome expression analysis.

#### 3.3.2. Involvement of Candidate Pathways in Gonadal Development and Gametogenesis

**Figure 6:**
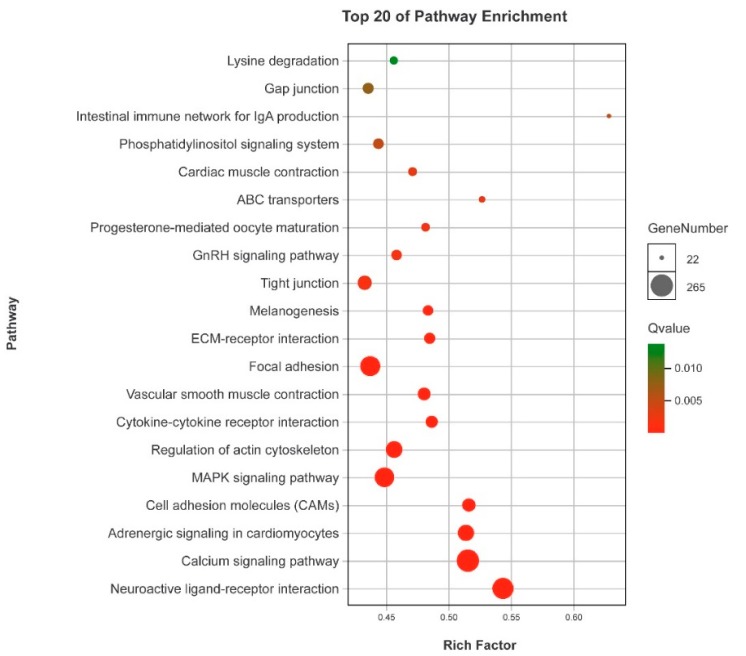
KEGG pathway enrichment analyses of DEGs.

In this study, we identified some enriched KEGG pathways related to silver sillago gonadal development and gametogenesis; i.e., neuroactive ligand-receptor interaction, calcium signaling pathway, adrenergic signaling in cardiomyocytes, cell adhesion molecules, MAPK signaling pathway, progesterone-mediated oocyte maturation, and GnRH signaling pathway (Figure 6). 

In teleost, it has been reported that the neuroactive ligand-receptor interaction pathway plays a vital role in teleost reproduction and gonadal development, such as in Nile tilapia [43], yellow perch [44] and spotted knifejaw [6] and olive flounder [9]. The GnRH signaling pathway plays an essential role in regulating the production and activating signal transduction cascades that ultimately modulate gonadotropin biosynthesis [45]. In this study, a large number of DEGs were found in this pathway, including growth hormone secretagogue receptor (*ghsr*), follicle stimulating hormone (*fsh*), gonadotropin-releasing hormone receptor (*gnrhr*), luteinizing hormone (*lhcgr*), thyroid stimulating hormone (*tsh*), and prostaglandin E receptor 1 (*ptger1*). These genes have been reported to be involved in the regulation of sex maturity, gonad development and reproduction by regulating the production and neuro-distribution of sex steroids [6].

It has been reported that Ca^2+^, PKCs and MAPKs may enable the gonadotrope to decode the nature of the pulsatile secretion of GnRH, and play an important role in the interaction network of the GnRHR [46]. Ca^2+^ and calmodulin kinase I and II participate in decoding GnRH pulse frequencies [47]. Calcium signaling plays a key role in male reproductive health. Several studies have shown that this pathway participates in cyclic adenosine monophosphate (cAMP)-induced steroidogenesis and male fertility [48,49,50]. Cell adhesion molecules play important roles in spermatogenesis in mice [51]. In fish, this pathway was found to be significantly enriched in the sex-biased genes [9]. The MAPK signaling pathway plays an important role in the regulation of gonadotropin subunit gene expression and the transition of primary spermatocytes across the blood-testis barrier [52]. In the crucifix crab, the MAPK signaling pathway was identified as one of the enriched pathways related to the reproduction [7]. In fish, the GnRH signaling pathway plays an essential role in regulating the production of and activating signal transduction cascades that ultimately modulate gonadotropin biosynthesis [45].

### 3.4. Discovery of Simple Sequence Repeats

**Figure 7:**
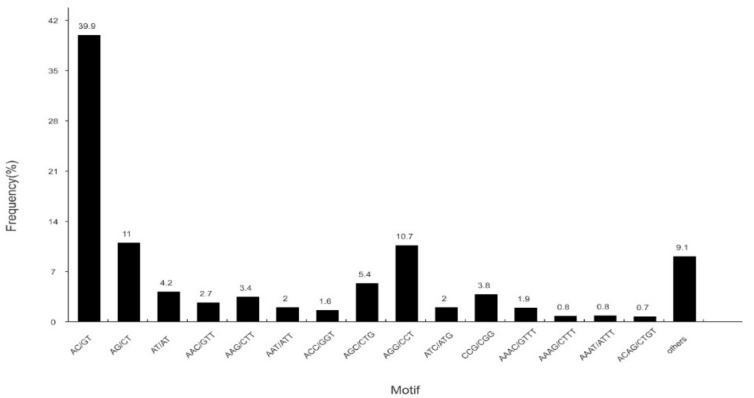
Distribution of identified single sequence repeats (SSRs) according to motif types in the sequences of *S. sihama*.

A total of 22,666 SSRs were identified in 14,577 SSR-containing sequences (Figure 7). Most SSR repeats were dinucleotide (12,579, 55.50%), followed by trinucleotide (7317, 32.28%), tetranucleotide (1871, 8.25%), pentanucleotide (500, 2.21%), and hexanucleotide (399, 1.76%). SSRs are important molecular markers that will serve as a valuable resource for parentage analysis, population genetics, and genetic linkage mapping in *S. sihama* in the future.

## 4. Conclusions

In conclusion, this work represents the first gonadal transcriptomes of *S. sihama* and 74,038 unigenes were generated. By comparing ovary and testis transcriptomes, numerous DEGs known to be involved in gonadal development and gametogenesis were identified. By KEGG enrichment analyses, the top 20 KEGG pathways with the highest number of DEGs were found to be pivotal in regulating gonadal development and gametogenesis in *S. sihama*. Moreover, 22,666 SSRs were identified in 14,577 SSR-containing sequences. These findings provide a valuable dataset for future functional analyses of sex-associated genes and molecular marker assisted selections in *S. sihama*.

## Figures and Tables

**Table 1:** Summary statistics of *Sillago sihama* gonad transcriptome sequencing data.

Sample^a^	Raw Reads	Clean Reads	Raw Bases	Clean Bases	Clean Reads Q20 (%)	Clean Reads Q30 (%)	Clean Reads Ratio (%)	GC Contents (%)
Ss_M	63,685,178	60,175,288	9,552,776,700	9,026,293,200	96.68	92.97	94.49	51.10
Ss_F	62,052,268	59,097,584	9,307,840,200	8,864,637,600	96.62	92.93	95.24	51.80

^a^ Male silver sillago named Ss_M; female silver sillago named Ss_F.

**Table 2:** *S. sihama* gonads transcriptome reference assembly and annotation statistics.

Database	Number
**Assembly**	
Number of unigenes	74,038
Max length (bp)	20,589
Min length (bp)	201
Average length (bp)	1,004
N50 (bp)	5,129
GC (%)	49.46
**Annotation**	
Total number of annotation unigenes	34,104
Unigene matches against NR	33,244
Unigene matches against Swissprot	28,924
Unigene matches against KOG	23,604
Unigene matches against KEGG	20,738
Unigene matches against GO	6,938

NR: Non-redundant; KOG: Eukaryotic orthologous group; KEGG: Kyoto Encyclopedia of Genes and Genomes; GO: Gene Ontology.

**Table 3:** List of putative genes significantly expressed in male and female gonads of *S. sihama*.

GeneID	Gene	log_2_ Ratio (Ss_F/Ss_M)	FDR
Unigene0025242	Anti-Mullerian hormone	−3.64	2.25 × 10^−20^
Unigene0033560	Androgen receptor-like	−2.93	3.95 × 10^−63^
Unigene0000289	Breast cancer anti-estrogen resistance 1	−2.68	2.17 × 10^−28^
Unigene0026099	Steroid 11-beta-hydroxylase	−9.94	4.27 × 10^−280^
Unigene0023574	Steroid 17-alpha-hydroxylase/17,20 lyase	−2.24	2.86 × 10^−75^
Unigene0038496	Sterol 26-hydroxylase, mitochondrial-like	−5.53	2.34 × 10^−279^
Unigene0029710	Doublesex- and mab-3-related transcription factor 1	−6.78	0
Unigene0040686	DM-related transcriptional factor Dmrt2b	−2.63	5.27 × 10^−141^
Unigene0006238	Doublesex- and mab-3-related transcription factor B1-like	−9.37	0
Unigene0039042	Estrogen receptor b1	−2.50	2.11 × 10^−106^
Unigene0012157	Forkhead box protein L1	−10.44	1.37 × 10 ^−14^
Unigene0026843	Follicle-stimulating hormone beta subunit	−4.42	2.03 × 10^−88^
Unigene0025516	Gonadal soma derived factor 1	−6.03	0
Unigene0015150	Insulin-like growth factor-binding protein 3	−4.32	7.62 × 10^−41^
Unigene0038426	Izumo sperm-egg fusion protein 1	−10.87	0
Unigene0062797	Ras-related and estrogen-regulated growth inhibitor	−4.35	4.21 × 10^−28^
Unigene0071926	R-spondin-1	−8.97	0.01
Unigene0026837	SRY-box containing gene 6b	−4.07	1.03 × 10^−07^
Unigene0039640	Transcription factor Sox-8	−4.58	6.84 × 10^−171^
Unigene0007384	Transcription factor Sox-10	−9.97	2.17 × 10^−08^
Unigene0014953	Sperm acrosome membrane-associated protein 6	−15.25	0
Unigene0041730	Sperm-associated antigen 17	−10.81	0
Unigene0035308	Spermatogenesis-associated protein 17	−5.50	0
Unigene0005539	Spermatogenesis-associated protein 4	−15.32	0
Unigene0038929	Sperm flagellar protein 2	−8.89	0
Unigene0026331	Breast cancer anti-estrogen resistance protein 3 homolog	2.91	1.32 × 10^−31^
Unigene0032976	Catenin beta-1	3.20	0
Unigene0052443	P450 aromatase	6.08	5.51 × 10^−19^
Unigene0040566	Doublesex- and mab-3-related transcription factor 3	4.50	0
Unigene0058550	Doublesex- and mab-3-related transcription factor A2	4.66	5.28 × 10^−37^
Unigene0021078	Estrogen-related receptor gamma-like	2.04	4.21 × 10^−08^
Unigene0021446	Factor in the germline alpha	3.79	9.67 × 10^−75^
Unigene0027009	Gonadotrophin releasing hormone receptor 1A	2.81	1.88 × 10^−47^
Unigene0040054	Gonadotropin-releasing hormone II receptor-like	5.99	2.84 × 10^−201^
Unigene0063145	Estradiol 17-beta-dehydrogenase 1	3.28	6.36 × 10^−15^
Unigene0016081	Insulin-like growth factor-binding protein 1	6.84	0
Unigene0028430	Oocyte-specific histone RNA stem-loop-binding protein 2-like	8.15	0
Unigene0005697	Sperm acrosome membrane-associated protein 4-like	14.78	4.34 × 10^−288^
Unigene0028731	Zona pellucida sperm-binding protein 2	10.99	0
Unigene0054018	Zona pellucida sperm-binding protein 3-like	10.64	0
Unigene0027464	Zona pellucida sperm-binding protein 4	11.61	0

FDR: False discovery rate.

## Supplementary Materials

The following are available online at http://www.mdpi.com/2073-4425/10/2/129/s1, Table S1: Primers used in real-time PCR, Table S2: 237 KEGG pathways in 9,806 unigenes, Table S3: Ss_M-vs-Ss_F GO Enrichment, Table S4: Top 27 KEGG pathways significantly enriched by DEGs, Table S5: Validation of the RNA-seq data by qRT-PCR, File S1: Scripts used for quality filtering, transcriptome assembly, and expression analyses.

## References

[1] Helfman G., Collette B.B., Facey D.E., Bowen B.W. (2009). The Diversity of Fishes: Biology, Evolution and Ecology.

[2] Janzen F.J. (1995). Experimental evidence for the evolutionary significance of temperature dependent sex determination. Evolution.

[3] Piferrer F. (2001). Endocrine sex control strategies for the feminization of teleost fish. Aquaculture.

[4] Heule C., Salzburger W., Böhne A. (2014). Genetics of sexual development: An evolutionary playground for fish. Genetics.

[5] Fan Z., You F., Wang L., Weng S., Wu Z., Zou Y., Tan X., Zhang P. (2014). Gonadal transcriptome analysis of male and female olive flounder (*Paralichthys olivaceus*). Biomed Res. Int..

[6] Du X., Wang B., Liu X., Liu X., He Y., Zhang Q., Wang X. (2017). Comparative transcriptome analysis of ovary and testis reveals potential sex-related genes and pathways in spotted knifejaw *Oplegnathus punctatus*. Gene.

[7] Zhang Y., Miao G., Fazhan H., Waiho K., Zheng H., Li S., Ikhwanuddin M., Ma H. (2018). Transcriptome-seq provides insights into sex-preference pattern of gene expression between testis and ovary of the crucifix crab (*Charybdis feriatus*). Physiol. Genomics.

[8] Yang D., Yin C., Chang Y., Dou Y., Hao Z., Ding J. (2016). Transcriptome analysis of male and female mature gonads of Japanese scallop *Patinopecten yessonsis*. Genes Genom..

[9] Zhang W., Liu Y., Yu H., Du X., Zhang Q., Wang X., He Y. (2016). Transcriptome analysis of the gonads of olive flounder (*Paralichthys olivaceus*). Fish Physiol. Biochem..

[10] Sun F., Liu S., Gao X., Jiang Y., Perera D., Wang X., Li C., Sun L., Zhang J., Kaltenboeck L., Dunham R., Liu Z. (2013). Male-biased genes in catfish as revealed by RNA-seq analysis of the testis transcriptome. PLoS ONE.

[11] Kaga T. (2013). Phylogenetic systematics of the family Sillaginidae (Percomorpha: Order Perciformes). Zootaxa.

[12] Gunn J.S., Milward N.E. (1985). The food, feeding habits and feeding structures of the whiting species *Sillago sihama* (Forsskål) and *Sillago analis* Whitley from Townsville, North Queensland, Australia. J. Fish Biol..

[13] Liu D., Guo Y., Wang Z., Liu C. (2012). Phylogenetics inferred from mitogenome and control region of Silver Sillago, *Sillago sihama*. Mitochondr. DNA.

[14] Shamsan E.F., Ansari Z.A. (2010). Studies on the reproductive biology of Indian Sand Whiting *Sillago sihama* (Forsskal). Indian J. Mar. Sci..

[15] Du T., Huang Y. (2009). Biological characteristics and indoor culture experiment in *Sillago sihama*. J. Aquac..

[16] Grabherr M.G., Haas B.J., Yassour M., Levin J.Z., Thompson D.A., Amit I., Adiconis X., Fan L. (2011). Trinity: reconstructing a full-length transcriptome without a genome from RNA-seq data. Nat. Biotechnol..

[17] Koonin E.V., Fedorova N.D., Jackson J.D., Jacobs A.R., Krylov D.M., Makarova K.S., Mazumder R., Mekhedov S.L., Nikolskaya A.N., Rao B.S. (2004). A comprehensive evolutionary classification of proteins encoded in complete eukaryotic genomes. Genome Biol..

[18] Mortazavi A., Williams B.A., McCue K., Schaeffer L., Wold B. (2008). Mapping and quantifying mammalian transcriptomes by RNA-seq. Nat. Methods.

[19] Mark D.R., Davis J.M., Gordon K.S. (2010). edgeR: A Bioconductor package for differential expression analysis of digital gene expression data. Bioinformatics..

[20] Ye J., Coulouris G., Zaretskaya I., Cutcutache I., Rozen S., Madden T. (2012). Primer-BLAST: A tool to design target-specific primers for polymerase chain reaction. BMC Bioinformatics.

[21] Zhang N., Du W.W., Wang Z.D., Huang Y., Du T., Dong Z.D. (2018). Screening of reference genes for Real-time PCR in different tissues from *Sillago sihama*. J. Guangdong Ocean University.

[22] Xu S., Xiao S., Zhu S., Zeng X., Luo J., Liu J., Gao T., Chen N. (2018). A draft genome assembly of the Chinese sillago (*Sillago sinica*), the first reference genome for Sillaginidae fishes. Gigascience..

[23] Yan H., Cui X., Shen X., Wang L., Jiang L., Liu H., Liu Y., Liu Q., Jiang C. (2018). De novo transcriptome analysis and differentially expressed genes in the ovary and testis of the Japanese mantis shrimp *Oratosquilla oratoria* by RNA-seq. Comp. Biochem. Phys. D..

[24] He L., Wang Q., Jin X., Wang Y., Chen L., Liu L., Wang Y. (2012). Transcriptome profiling of testis during sexual maturation stages in *Eriocheir sinensis* using Illumina sequencing. PLoS ONE.

[25] Ma K., Qiu G., Feng J., Li J. (2012). Transcriptome analysis of the oriental river prawn, *Macrobrachium nipponense* using 454 pyrosequencing for discovery of genes and markers. PLoS ONE.

[26] Meng X.L., Liu P., Jia F.L., Li J., Gao B.Q. (2015). De novo transcriptome analysis of *Portunus trituberculatus* ovary and testis by RNA-seq: identification of genes involved in gonadal development. PLoS ONE.

[27] Tilmann C., Capel B. (2002). Cellular and molecular pathways regulating mammalian sex determination. Recent Prog. Horm. Res..

[28] Webster K.A., Schach U., Ordaz A., Steinfeld J.S., Draper B.W., Siegfried K.R. (2017). Dmrt1 is necessary for male sexual development in zebrafish. Dev. Biol..

[29] Pfennig F., Standke A., Gutzeit H.O. (2015). The role of Amh signaling in teleost fish—Multiple functions not restricted to the gonads. Gen. Comp. Endocrinol..

[30] Zhai G., Shu T., Xia Y., Jin X., He J.Y., Zhan Y. (2017). Androgen signaling regulates the transcription of anti-Müllerian hormone via synergy with SRY-related protein SOX9A. Sci. Bull..

[31] Sawatari E., Shikina S., Takeuchi T., Yoshizaki G. (2007). A novel transforming growth factor-β superfamily member expressed in gonadal somatic cells enhances primordial germ cell and spermatogonial proliferation in rainbow trout (*Oncorhynchus mykiss*). Dev. Biol..

[32] Kaneko H., Ijiri S., Kobayashi T., Izumi H., Kuramochi Y., Wang D.S., Mizuno S., Nagahama Y. (2015). Gonadal soma-derived factor (gsdf), a TGF-β superfamily gene, induces testis differentiation in the teleost fish *Oreochromis niloticus*. Mol. Cell Endocrinol..

[33] Svechnikov K., Söder O. (2008). Ontogeny of gonadal sex steroids. Best Pract. Res. Cl. En..

[34] Applebaum S.L., Wilson C.A., Holt G.J., Nunez B.S. (2010). The onset of cortisol synthesis and the stress response is independent of changes in *CYP11B* or *CYP21* mRNA levels in larval red drum (*Sciaenops ocellatus*). Gen. Comp. Endocr..

[35] Socorro S., Martins R.L., Mylonas C., Canario A.V. (2007). A cDNA for European sea bass (*Dicentrachus labrax*) 11β-hydroxylase: gene expression during the thermosensitive period and gonadogenesis. Gen. Comp. Endocrinol..

[36] Liu S., Govoroun M., D’Cotta H., Ricordel M.J., Lareyre J.J., McMeel O.M., Smith T., Nagahama Y., Guiguen Y. (2000). Expression of cytochrome p450 (11β-hydroxylase) gene during gonadal sex differentiation and spermatogenesis in rainbow trout, *Oncorhynchus mykiss*. J. Steroid. Biochem Mol. Biol..

[37] Li Q., Du X., Pan Z., Zhang L., Li Q. (2018). The transcription factor SMAD4 and miR-10b contribute to E2 release and cell apoptosis in ovarian granulosa cells by targeting CYP19A1. Mol. Cell Endocrinol..

[38] Yamashita A., Ito M., Takamatsu N., Shiba T. (2000). Characterization of solt, a novel SoxLZ/Sox6 binding protein expressed in adult mouse testis. Febs. Lett..

[39] Barrionuevo F., Scherer G. (2010). SOX E genes: SOX9 and SOX8 in mammalian testis development. Int. J. Biochem. Cell B..

[40] Lorenzetti D., Poirier C., Zhao M., Overbeek P.A., Harrison W., Bishop C.E. (2014). A transgenic insertion on mouse chromosome 17 inactivates a novel immunoglobulin superfamily gene potentially involved in sperm-egg fusion. Mamm. Genome..

[41] Zeng S., Gong Z. (2002). Expressed sequence tag analysis of expression profiles of zebrafish testis and ovary. Gene.

[42] Liu X., Wang H., Gong Z. (2006). Tandem-repeated zebrafish *zp3* genes possess oocyte-specific promoters and are insensitive to estrogen induction1. Biol. Reprod..

[43] Sun L.X., Wang Y.Y., Zhao Y., Wang H., Li N., Ji X.S. (2016). Global DNA methylation changes in *Nile tilapia* gonads during high temperature-induced masculinization. PLoS ONE.

[44] Li Y.H., Wang H.P., Yao H., O’Bryant P., Rapp D., Guo L., Waly E.A. (2017). De novo transcriptome sequencing and analysis of male, pseudo-male and female yellow perch, *Perca flavescens*. PLoS ONE.

[45] Ruf F., Sealfon S.C. (2004). Genomics view of gonadotrope signaling circuits. Trends Endocrin. Met..

[46] Naor Z. (2009). Signaling by G-protein-coupled receptor (GPCR): studies on the GnRH receptor. Front. Neuroendocrin..

[47] Ferris H.A., Shupnik M.A. (2006). Mechanisms for pulsatile regulation of the gonadotropin subunit genes by GNRH11. Biol. Reprod..

[48] Sullivan M.H., Cooke B.A. (1986). 1986 The role of Ca^2+^ in steroidogenesis in Leydig cells. Stimulation of intracellular free Ca^2+^ by lutropin (LH), luliberin (LHRH) agonist and cyclic AMP. Biochem. J..

[49] Lee J.H., Ahn H.J., Lee S.J., Gye M.C., Min C.K. (2011). Effects of L- and T-type Ca^2+^ channel blockers on spermatogenesis and steroidogenesis in the prepubertal mouse testis. J. Assist. Reprod. Genet..

[50] Abdou H.S., Villeneuve G., Tremblay J.J. (2013). The calcium signaling pathway regulates Leydig cell steroidogenesis through a transcriptional cascade involving the nuclear receptor NR4A1 and the steroidogenic acute regulatory protein. Endocrinology.

[51] Pelz L., Purfürst B., Rathjen F.G. (2017). The cell adhesion molecule BT-IgSF is essential for a functional blood-testis barrier and male fertility in mice. J. Biol. Chem..

[52] Di A.S., Rossi P., Geremia R., Sette C. (2002). The MAPK pathway triggers activation of Nek2 during chromosome condensation in mouse spermatocytes. Development.

