# Supplementary material for: Transcriptome Analysis of Male and Female Mature Gonads of Silver Sillago (Sillago sihama)
Source: Genes (Basel). 2019 Feb 11;10(2):129. doi: 10.3390/genes10020129 (PMC6409516; doi:10.3390/genes10020129)
Supplement: Supplementary file 1 [file genes-10-00129-s001.zip › Supplementary Materials/Table S1 Primers used in real-time PCR.docx]

Table S1. Primers used in real-time PCR.

| Gene | Forward Primer (5'-3') | Reverse Primer (5'-3') | Product, bp | Tm, ℃ |
| --- | --- | --- | --- | --- |
| *amh* | TCCAAACACGGCCAACATCA | GGAGAGGTAAGTCCCGTGTC | 184 | 60 |
| *cyp11b* | CTCGGAGAGGACCTTCAACG | CGCCGTTCTTGAGGAAGACT | 203 | 60 |
| *cyp27a1* | CAGGAGTCGACACAACCTCC | GGATACAGCCGTAGCGTCTC | 190 | 60 |
| *dmrt1* | TCCGCCCTGAAGGGACACAA | GGACAGAGCCACTGGACTACAA | 165 | 60 |
| *dmrtb1* | CGTGTCCCAACTTCATGCTCA | CCACAAGCCGCCTCGAAT | 165 | 60 |
| *foxl1* | CAAAGTCGCTGGACACAAGC | GGCTGCTCAGGTTGGTTCAA | 180 | 60 |
| *gsdf* | GGCTCTTTGGTGACGATTGC | CTTGTCTCGGTGAGCGATGT | 234 | 60 |
| *izumo1* | TCGAGACCGAAAGGGAGTCA | TCTCTAAGATCCTCCTGCCCT | 155 | 60 |
| *cyp19a1a* | GGGGCCGCTTCTGTCATATT | ATGCCTCTCTCGTTCATGCC | 228 | 60 |
| *dmrt3* | GTACCTGTACATGGGGGCTC | AGGATGCATTTCTCGCAGGT | 156 | 60 |
| *gnrhr2* | GTGGAACATCACCGTCCAGT | CTGATGGCCAGAGGGTTGAG | 150 | 60 |
| *igfbp1* | CAGAGGGCCTTGGACAAGAT | AGGAGTTCACGCACCAACAT | 160 | 60 |
| *spaca4* | GTCATGTCTTGTCGGGGTGT | TGAACCCCAAAGCTCCTCAC | 157 | 60 |
| *zp2* | TCCCCAATGGAACCATGACG | CAGCCGAGTTTGATCCCCTT | 246 | 60 |
| *zp4* | CAGGGACAGCCATTTCGAGT | GATTGGGTAGTCGGCTGGAG | 223 | 60 |
| *rpl7* | GCAAAGTGACCAGGAAACTGAT | GGCTGACACCGTTGATACCTCT | 180 | 60 |
