# Supplementary material for: Transcriptome Analysis of Male and Female Mature Gonads of Silver Sillago (Sillago sihama)
Source: Genes (Basel). 2019 Feb 11;10(2):129. doi: 10.3390/genes10020129 (PMC6409516; doi:10.3390/genes10020129)
Supplement: Supplementary file 1 [file genes-10-00129-s001.zip › Supplementary Materials/Table S4 Top 27 KEGG pathways significantly enriched by DEGs.docx]

Table S4. Top 27 KEGG pathways significantly enriched by DEGs

| Pathway | DEGs with Pathway Annotation | Q value | Pathway ID |
| --- | --- | --- | --- |
| Neuroactive ligand-receptor interaction | 249 (7.49%) | 5.81E-18 | ko04080 |
| Calcium signaling pathway | 265 (7.97%) | 2.13E-15 | ko04020 |
| Adrenergic signaling in cardiomyocytes | 183 (5.51%) | 2.39E-10 | ko04261 |
| Cell adhesion molecules (CAMs) | 142 (4.27%) | 3.40E-08 | ko04514 |
| MAPK signaling pathway | 228 (6.86%) | 3.72E-06 | ko04010 |
| Regulation of actin cytoskeleton | 189 (5.69%) | 9.72E-06 | ko04810 |
| Vascular smooth muscle contraction | 136 (4.09%) | 1.15E-05 | ko04270 |
| Cytokine-cytokine receptor interaction | 127 (3.82%) | 1.15E-05 | ko04060 |
| Focal adhesion | 234 (7.04%) | 1.73E-05 | ko04510 |
| ECM-receptor interaction | 113 (3.4%) | 4.85E-05 | ko04512 |
| Melanogenesis | 104 (3.13%) | 1.28E-04 | ko04916 |
| Progesterone-mediated oocyte maturation | 79 (2.38%) | 1.59E-03 | ko04914 |
| GnRH signaling pathway | 105 (3.16%) | 1.59E-03 | ko04912 |
| Tight junction | 155 (4.66%) | 1.59E-03 | ko04530 |
| ABC transporters | 49 (1.47%) | 1.99E-03 | ko02010 |
| Cardiac muscle contraction | 82 (2.47%) | 2.47E-03 | ko04260 |
| Phosphatidylinositol signaling system | 107 (3.22%) | 4.95E-03 | ko04070 |
| Intestinal immune network for IgA production | 22 (0.66%) | 5.09E-03 | ko04672 |
| Gap junction | 112 (3.37%) | 7.67E-03 | ko04540 |
| Lysine degradation | 73 (2.2%) | 1.37E-02 | ko00310 |
| Inositol phosphate metabolism | 76 (2.29%) | 1.41E-02 | ko00562 |
| Phospholipase D signaling pathway | 6 (0.18%) | 1.49E-02 | ko04072 |
| Dorso-ventral axis formation | 31 (0.93%) | 1.56E-02 | ko04320 |
| Glycosphingolipid biosynthesis - lacto and neolacto series | 22 (0.66%) | 1.82E-02 | ko00601 |
| Glycerophospholipid metabolism | 73 (2.2%) | 2.02E-02 | ko00564 |
| Purine metabolism | 133 (4%) | 2.38E-02 | ko00230 |
| Phototransduction | 24 (0.72%) | 4.46E-02 | ko04744 |
