# Supplementary material for: Transcriptome Analysis of Male and Female Mature Gonads of Silver Sillago (Sillago sihama)
Source: Genes (Basel). 2019 Feb 11;10(2):129. doi: 10.3390/genes10020129 (PMC6409516; doi:10.3390/genes10020129)
Supplement: Supplementary file 1 [file genes-10-00129-s001.zip › Supplementary Materials/Table S5 Validation of the RNA-seq data by qRT-PCR.docx]

Table S5**.** Validation of the RNA-seq data by qRT-PCR.

| **GeneID** | **Gene** | **RNA-Seq** | |  | **qRT-PCR** | |
| --- | --- | --- | --- | --- | --- | --- |
|  |  | **Log_2_FC (female/male)** | **P-value** |  | **Log_2_FC (female/male)** | **P-value** |
| Unigene0025242 | *amh* | -3.64 | 2.25E-20 |  | -1.68 | 0.00178645 |
| Unigene0026099 | *cyp11b* | -9.94 | 4.27E-280 |  | -3.23 | 5.21E-07 |
| Unigene0038496 | *cyp27a1* | -5.53 | 2.34E-279 |  | -7.71 | 3.92665E-06 |
| Unigene0029710 | *dmrt1* | -6.78 | 0 |  | -2.02 | 1.8665E-07 |
| Unigene0006238 | *dmrtb1* | -9.37 | 0 |  | -2.46 | 2.7148E-09 |
| Unigene0012157 | *foxl1* | -10.44 | 1.37E-14 |  | -4.43 | 4.1801E-07 |
| Unigene0025516 | *gsdf* | -6.03 | 0 |  | -2.9 | 6.8479E-06 |
| Unigene0038426 | *izumo1* | -10.87 | 0 |  | -2.97 | 0.00076591 |
| Unigene0052443 | *cyp19a1a* | 6.08 | 5.51E-19 |  | 4.38 | 0.00033372 |
| Unigene0040566 | *dmrt3* | 4.5 | 0 |  | 4.31 | 5.0041E-05 |
| Unigene0040054 | *gnrhr2* | 5.99 | 2.84E-201 |  | 8.88 | 4.0098E-05 |
| Unigene0016081 | *igfbp1* | 6.84 | 0 |  | 4.79 | 2.982E-08 |
| Unigene0005697 | *spaca4* | 14.78 | 4.34E-288 |  | 8.86 | 0.00011742 |
| Unigene0028731 | *zp2* | 10.99 | 0 |  | 15.58 | 0.00020059 |
| Unigene0027464 | *zp4* | 11.61 | 0 |  | 16.35 | 0.00013064 |
