# Supplementary material for: Transcriptome Analysis of Male and Female Mature Gonads of Silver Sillago (Sillago sihama)
Source: Genes (Basel). 2019 Feb 11;10(2):129. doi: 10.3390/genes10020129 (PMC6409516; doi:10.3390/genes10020129)
Supplement: Supplementary file 1 [file genes-10-00129-s001.zip › Supplementary Materials/File S1. Scripts used for quality filtering, transcriptome assembly, and expression analyses.doc]

S1. Supplementary information: Scripts used for quality filtering, transcriptome assembly, and expression analyses.

**Raw Reads Filtering**

Reads obtained from the sequencing machines included dirty reads containing adapters or low quality bases which would affect the following assembly and analysis. Thus, to get high quality clean reads, reads were further filtered according to the following rules by Trimmomatic software:

1) Remove adapters;
2) Remove leading low quality or N bases (below quality 3);
3) Remove trailing low quality or N bases (below quality 3);
4) Scan the read with a 4-base wide sliding window, cutting when the average quality per base drops below 15;
5) Drop reads below the 36 bases long.

**Trimmomatic Code:**

bsub -q long -n 10 -o ./ java -jar /gpfs0/home/tzz0016/trimmomatic/Trimmomatic-0.36/trimmomatic-0.36.jar PE -phred33 /gpfs0/home/tzz0016/ raw_reads/Ss_female_1.fastq /gpfs0/home/tzz0016/changxu_sex/raw_reads/Ss_female_2.fastq trimmed_Ss_female_1_paired.fq trimmed_Ss_female_1_unpaired.fq trimmed_Ss_female_2_paired.fq trimmed_Ss_female_2_unpaired.fq ILLUMINACLIP:/gpfs0/home/tzz0016/trimmomatic/Trimmomatic-0.36/adapters/TruSeq3-PE.fa:2:30:10 LEADING:3 TRAILING:3 SLIDINGWINDOW:4:15 MINLEN:36

bsub -q long -n 10 -o ./ java -jar /gpfs0/home/tzz0016/trimmomatic/Trimmomatic-0.36/trimmomatic-0.36.jar PE -phred33 /gpfs0/home/tzz0016/ raw_reads/Ss_male_1.fastq /gpfs0/home/tzz0016/changxu_sex/raw_reads/Ss_male_2.fastq trimmed_Ss_male_1_paired.fq trimmed_Ss_male_1_unpaired.fq trimmed_Ss_male_2_paired.fq trimmed_Ss_male_2_unpaired.fq ILLUMINACLIP:/gpfs0/home/tzz0016/trimmomatic/Trimmomatic-0.36/adapters/TruSeq3-PE.fa:2:30:10 LEADING:3 TRAILING:3 SLIDINGWINDOW:4:15 MINLEN:36

***De novo* Assembly**

Transcriptome *de novo* assembly was carried out with short reads assembling program – Trinity by default parameters.

**Trinity code:**

bsub -q bigmem -n 16 -o ./ Trinity --seqType fq --max_memory 200G --CPU 16 -min_kmer_cov 3 –left /gpfs0/home/tzz0016/ trimmed_Ss_female_1.fq,/gpfs0/home/tzz0016/ trimmed_Ss_male_1.fq, --right /gpfs0/home/tzz0016/ trimmed_Ss_female_2.fq,/gpfs0/home/tzz0016/ trimmed_Ss_male_2.fq

**Alignment with reference transcriptome and quantification of Gene Abundance**

High quality clean reads were mapped to the reference transcriptome using short reads alignment tool Bowtie2 by default parameters. The gene abundances were calculated and normalized to RPKM (Reads Per kb per Million reads)

**Bowtie2 code:**

Bowtie2 -x Ss_gonad_RNAseq -1 /gpfs0/home/tzz0016/ trimmed_Ss_female_1.fastq -2 /gpfs0/home/tzz0016/ trimmed_Ss_female_2.fastq > Ss_female.sam

samtools view -@ 20 -S -b -u -T Ss_gonad_RNAseq.fasta Ss_female.sam > Ss_female.bam

samtools sort -@ 20 Ss_female.bam Ss_female_sorted

samtools index Ss_female_sorted.bam

samtools idxstats Ss_female_sorted.bam > Ss_female.txt

Bowtie2 -x Ss_gonad_RNAseq -1 /gpfs0/home/tzz0016/ trimmed_Ss_male_1.fastq -2 /gpfs0/home/tzz0016/ trimmed_Ss_male_2.fastq > Ss_male.sam

samtools view -@ 20 -S -b -u -T Ss_gonad_RNAseq.fasta Ss_male.sam > Ss_male.bam

samtools sort -@ 20 Ss_male.bam Ss_male_sorted

samtools index Ss_male_sorted.bam

samtools idxstats Ss_male_sorted.bam > Ss_male.txt

**Analysis of Differentially expressed genes (DEGs)**

To identify differentially expressed genes across samples or groups, the edgeR package (http://www.r-project.org/) was used. We identified genes with a fold change ≥4 and a false discovery rate (FDR) <0.05 in a comparison as significant DEGs.

**edgeR Code:**

setwd("D:/R/gonad")

library(edgeR)

x <- read.delim("gonad_RNA_seq_reads_mapping_sillago_CDS.txt", row.names="gene")

dim(x)

head(x)

group <- factor(c(1,1,2,2,3,3,4,4,5,5,6,6))

\# Make a DGEList object from the data table (x)

y <- DGEList(counts=x, group=group)

dim(y)

#sample information

y$samples

#Plot samples on a two-dimensional scatterplot so that distances on the plot approximate the typical log2 fold changes between the samples

plotMDS(y)

#plot library size

barplot(y$samples$lib.size*1e-6,names=c("Ss_female","Ss_male"), ylab="Library size (millions)")

#filter out lowly expressed genes

keep <- rowSums(cpm(y)>1) >= 2

y <- y[keep, , keep.lib.sizes=FALSE]

dim(y)

#Normalization

\# In edgeR, normalization takes the form of correction factors that enter into the statisticalmodel. Such correction factors are usually computed internally by edgeR functions, but it is also possible for a user to supply them. users should not enter RPKM or FPKM values to edgeR in place of read counts.

y <- calcNormFactors(y)

\# Statistical Model

design <- model.matrix(~group)

\# To estimate common dispersion

y <- estimateDisp(y,design)

\# Generalized linear model

fit <- glmFit(y, design)

\# To compare 2 VS 1

lrt.2vs1 <- glmLRT(fit, coef=2)

topTags(lrt.2vs1)

results <- (topTags(lrt.2vs1, n=nrow(lrt.2vs1$table) )$table)

head(results)

write.csv(results, "edgeR.ExactTest.male_VS_female.csv")

\# Testing for DE genes

et <- exactTest(y)

topTags(et)

results <- (topTags(et, n=nrow(et$table) )$table)

head(results)

write.csv(results, "edgeR.ExactTest.Results.csv")
